# Postnatal growth of small for gestational age late preterm infants: determinants of catch-up growth

**DOI:** 10.1038/s41390-022-02402-3

**Published:** 2022-12-02

**Authors:** Giulia Vizzari, Daniela Morniroli, Valentina Tiraferri, Marta Macchi, Silvana Gangi, Alessandra Consales, Federica Ceroni, Jacopo Cerasani, Fabio Mosca, Maria Lorella Giannì

**Affiliations:** 1 Fondazione IRCCS Ca’ Granda Ospedale Maggiore Policlinico, NICU, Milan, Italy; 2 Department of Clinical Sciences and Community Health, University of Milan, Milan, Italy

## Abstract

**Background:**

Failure to recover growth is a risk reported in late preterm population. This study aimed to evaluate the auxological outcome of late preterm infants and identify factors associated with failure to recover growth.

**Methods:**

We enrolled late preterm infants with birth weight ≤10th percentile, followed up at High-Risk Infant Follow-up Service. We collected data at birth and at follow-up visits. A logistic regression analysis was performed to assess variables independently associated with growth failure.

**Results:**

The population consisted of 175 preterms. The percentage of children showing no weight recovery was 34% at 36 months. At logistic regression analysis, infants who had not regained weight at 12 months had a higher risk of not regaining weight even at 36 months. The same risk factor was highlighted for length catch-up growth. Moreover, infants fed any human milk at discharge were protected from not achieving both weight and length catch-up growth at 36 months.

**Conclusion:**

These results indicate that children born late preterm and small for gestational age could fail to recover weight and stature growth in the first 36 months. The protective effect of human milk on failure to thrive highlights the importance of promoting breastfeeding in this population.

**Impact:**

- A significant number of SGA late preterms show a failure to recover weight and statural growth.
- Having experienced intrauterine growth restriction is associated with a greater chance of achieving statural catch-up growth.
- Being born singleton represents a risk factor for slower weight and height growth velocity.
- Breastmilk has a protective effect on failure to recover adequate weight and length in preterm SGA infants. This finding highlights the importance of promoting breastfeeding in this population.

## Introduction

Late preterm infants, defined as newborns born between 34 0/7 and 36 6/7 weeks of gestational age, have emerged in the last decade as a new high-risk group in neonatology due to their physiological and structural immaturity.^1,2^ Increasing evidence has indicated that late preterm infants show higher mortality and morbidity occurrence both during the hospital stay and in the long term compared to term infants.^1,3^ Moreover, an increased risk for postnatal growth restriction up to preschool age has been detected among late preterm infants, probably due to inadequate nutrition and the experience of feeding difficulties.^4^ Infants born small for gestational age (SGA) are even at higher risk for postnatal growth retardation.^4,5^ Remarkably, within such a complex context, despite the well-known health benefits associated with human milk feeding, concerns remain about SGA infants’ growth when they are human milk-fed.^5^

There is a paucity of data concerning the neonatal variables and the mode of feeding associated with the persistence of postnatal growth retardation, defined as having a weight, length and head circumference below the 10th percentile in SGA late preterm infants.

This study aimed to assess the persistence of postnatal growth retardation in the first 36 months of age in a cohort of SGA late preterm infants and to evaluate the neonatal variables and the mode of feeding during hospital stay associated with its persistency.

## Methods

We conducted a retrospective study including preterm infants born between 2009 and 2015 admitted to the authors’ institution, participating in our post-discharge follow-up program. According to the Fenton growth charts, the inclusion criteria were gestational age between 34 and 36 weeks and birth weight <10th percentile.^6–8^ Exclusion criteria were congenital diseases, chromosomal abnormalities, severe cardiac, brain, renal, endocrinologic and gastrointestinal diseases that could interfere with growth.

Parents prospectively consented to the use of the anonymized data for research purposes. The Ethics Committee of the Fondazione IRCCS Ca’ Granda Ospedale Maggiore Policlinico approved the retrospective study.

### Data collection

The neonatal, clinical and feeding data during hospital stay were collected from the patient’s computerized medical charts. The neonatal data included gestational age, anthropometric parameters at birth (weight, length and head circumference), sex, being twin, mode of delivery, length of hospitalization, ethnicity, obstetric history of intrauterine growth restriction (IUGR) based on a combination of measures of fetal size percentile and Doppler abnormalities.^9^ In addition, maternal data were further collected, including age, education, assisted conception and occurrence of preeclampsia, defined as de novo hypertension after 20 weeks gestation with new-onset proteinuria.^10^

The medical data recorded were the following: Apgar score at 1 and 5 min, need for respiratory support, the occurrence of jaundice requiring phototherapy and/or hypoglycemia defined as a plasma glucose level <45 mg/dl and/or perinatal infection defined by the presence of positive blood culture and/or by clinical and laboratory signs, mode of feeding at discharge (exclusively human milk feeding, mixed feeding including the use of both human milk and formula, exclusively formula feeding).

Post-discharge anthropometric parameters (weight, length and head circumference) were collected from patients’ follow-up medical charts at 3, 6, 12 and 24 months of corrected age (calculated from the chronologic age, adjusting for gestational age) and 36 months of chronological age. According to the World Health Organization growth charts, catch-up growth at 12, 24 and 36 months was defined as the achievement of a percentile ≥10th either for weight or length or head circumference.^11^

### Nutritional practices

Regarding late preterm infants, enteral feeding was started within the first 24 h of life in all newborns who were in stable clinical conditions. Fresh mother’s milk represented the first choice and mothers were encouraged to directly breastfed their infants or, when this was not possible, to express their milk soon after birth. Furthermore, the health care professional supported breastfeeding in agreement with the cue-based feeding method during the hospital stay.^12,13^

According to the nutritional procedure of our Center, human milk was fortified with bovine milk-based fortifiers in all newborns with weight ≤1800 g and with enteral intake ≥80 ml/kg/day with breastmilk for more than 50% of the total intake. If breastmilk was insufficient or not available, formula milk feeding was started.

### Statistical analysis

Continuous variables were presented as mean values and standard deviation (SD). Categorical variables were expressed as numbers (frequencies).

To identify the independent variables associated with the absence of weight and length catch-up growth at 36 months of age, a multivariate binary logistic regression analysis was performed. The independent variables entered in the model were sex, need for respiratory support, the occurrence of infection, obstetrical history of intrauterine growth retardation, absence of catch-up growth at 12 months of corrected age, being a twin and milk feeding grouped as any human milk vs exclusively formula feeding.

Statistical analyses were made using SPSS (Statistical Package for the Social Sciences) version 22 software (SPSS Inc., Chicago, IL).

## Results

A total of 175 infants were enrolled, and their basic characteristics are reported in Table 1. Length and head circumference at birth were available for 109 and 106 infants, respectively. Mothers’ basic characteristics are also reported in Table 1.

**Table 1:** Basic infants’ and mothers’ characteristics.

Infants’ and mothers’ characteristics	
*Infants, n* *=* *175*	Mean (SD)
Gestational age (weeks)	35.2 ± 0.7
Birth weight (g)	1772 (119)
Birth length (cm)	43.1 (2.1)
Birth head circumference (cm)	30.8 (1.1)
Apgar score 1’	8 (1.0)
Apgar score 5’	9 (0.7)
	*N* (%)
Birth weight ≤10 and ≥3 pct	100 (57)
Birth weight <3 pct	75 (43)
Birth length ≤10 and ≥3 pct	17 (16)
Birth length <3 pct	41 (38)
Birth head circumference ≤10 and ≥3 pct	19 (18)
Birth head circumference <3 pct	23 (22)
Males	81(46)
Twins	97 (55)
Intrauterine growth retardation	91 (52)
Ethnicity	
Caucasian	154 (88)
Black	6 (3)
Asian	6 (3)
Hispanic	9 (6)
Mode of delivery	
Caesarean section	154 (88)
Vaginal delivery	21 (12)
*Mothers, n* *=* *167*	
Age (years)	
<30	19 (11)
30–45	140 (84)
>45	8 (5)
Education (years)	
≤13	74 (44)
>13	93 (56)
Assisted conception	59 (35)
Preeclampsia	59 (35)
Multiple pregnancy	90 (54)

*Pct* percentile.

Length of hospital stay was between 11 and 30 days in 76% of cases, whereas 19% and 5% of infants were discharged before ten days and after 30 days, respectively. During the hospital stay, 45% of infants developed jaundice requiring phototherapy, 9% showed hypoglycemia, 12.6% required respiratory support, and 5% developed a perinatal infection. At discharge, 18% and 36% of infants were exclusively human milk-fed and complementary fed, respectively, whereas 46% were exclusively formula-fed.

**Fig. 1:**
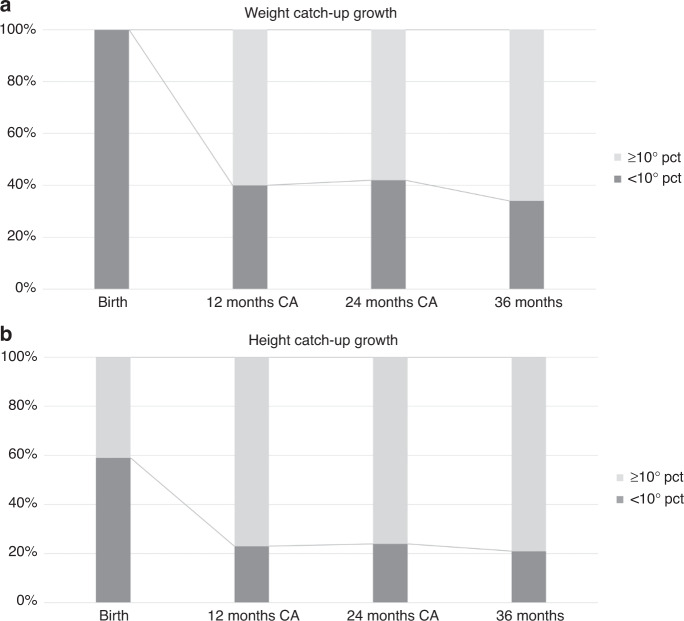
Weight and height catch-up growth. Percentage of infants showing weight and height percentiles <10 and ≥10. CA corrected age, Pct percentile.

At 3 and 6 months of corrected age, 85% and 79% of infants showed a weight catch-up growth, respectively. Concerning length, 84% and 86% of infants had a length percentile above or equal to the 10th percentile at 3 and 6 months, respectively, whereas head circumference catch-up growth was already achieved by 95% of infants at both time points. At the following study time points, the percentage of infants who did show a weight equal to or above the 10th percentile decreased to 60% at 12 and 24 months, achieving 75% at 36 months. Regarding length, the percentage of infants who achieved catch-up growth was around 79% through the subsequent time points (Fig. 1). Head circumference catch-up growth was achieved by most infants already at 12 and 24 months.

**Fig. 2:**
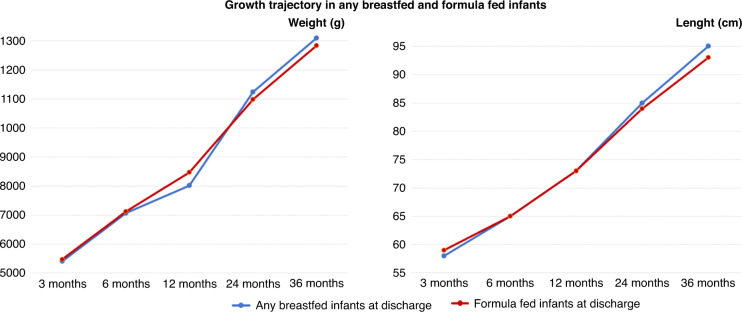
Growth trajectory. Mean growth trajectory in any breastfed and formula-fed infants at each time point.

In Fig. 2 the mean growth trajectories of any breastfed and exclusively formula-fed infants at each time point of the study are shown.

In a multivariate binary logistic regression analysis, infants who had not caught up about weight at 12 months and were born singletons were at higher risk for not showing weight catch-up growth at 36 months. Regarding length, infants who had not achieved a length catch-up growth at 12 months, were born singletons and had no intrauterine growth restriction (IUGR) were at higher risk for not having length catch-up growth at 36 months. Infants fed any human milk were protected from not achieving both weight and length catch-up growth at 36 months (Table 2).

**Table 2:** Variables independently associated with the absence of weight and length catch-up growth at 36 months of age at multivariate binary logistic regression.

	Absence of weight catch-up growth			Absence of length catch-up growth		
	OR	95% IC	*P* value	OR	95% IC	*P* value
Gender (male vs female)	1.01	0.48–2.17	0.9	0.84	0.31–2.25	0.73
Respiratory support (yes vs no)	1.63	0.49–5.40	0.4	0.85	0.18–3.99	0.84
Perinatal infection (yes vs no)	1.39	0.27–7.10	0.7	1.22	0.15–9.60	0.84
Being twin (no vs yes)	1.61	1.08–2.40	0.02	1.88	1.11–3.18	0.018
Weight <10 pct at 12 months (yes vs no)	9.31	4.28–20.28	<0.001	34.65	11.46–104.77	<0.001
Any human milk feeding during hospital stay (yes vs no)	0.59	0.40–0.9	0.011	0.57	0.33–0.99	0.046
Intrauterine growth restriction (no vs yes)	1.04	0.49–2.23	0.90	2.98	1.06–8.38	0.038

*Pct* percentile.

## Discussion

The literature shows that being a late preterm neonate represents a risk factor for adverse short and long-term outcomes compared to full-term newborns.^14^ However, because of a paucity of data regarding the neonatal variables and the mode of feeding associated with the persistence of postnatal growth restriction, catch-up growth represents one of the main challenges for clinicians.

According to our findings, more than 80% of our late preterm and SGA infants showed a catch-up growth in weight and length at 3 and 6 months of corrected age. On the other hand, weight growth tended to decrease, and almost 60% of children maintained to catch-up growth at 12 and 24 months of corrected age, while the growth trajectory for length remained substantially stable for 70% of children. The decrease in the growth trajectory following the first 6 months of corrected age could be partially explained by the introduction of solid foods between 5 and 8 months of life.^15^ Indeed, complementary feeding in preemies represents a crucial period that requires the acquisition of specific neurodevelopmental skills and that is linked to the onset of new symptoms related to oromotor dysfunction and avoidant feeding behavior, such as choking and spitting.^4,15^ Furthermore, two surveys conducted in Italy and UK underlined that many mothers of low birth weight newborns, with the introduction of complementary feeding, didn’t perceive the importance of an adequate energy intake and offered their infants nutritionally inadequate diets.^16,17^

At 36 months of corrected age, however, only one-third of children showed weight and length values lower than the 10th percentile, highlighting a variety of growth trajectories during early infancy. Moreover, not having reached catch-up growth at 12 months and being born singleton were associated with an increased risk of not reaching both weight and length catch-up growth even at 36 months.

On the other hand, the vast majority of our cohort already achieved head circumference catch-up growth at 12 months. The importance of this result is emphasized by the close relationship between head circumference growth and neurodevelopmental outcome of preterm infants, as described in the literature.^18,19^

There is a lack of data regarding the weight and length growth trajectory of late preterm infants. Zhang et al. in a recent observational study, evaluated the growth in a cohort of 599 healthy late preterm infants and showed a catch-up growth in length and weight of 30.7% and 46.2%, respectively, at term corrected age, but no data on early childhood growth were available.^20^ Han et al., in their longitudinal study, assessed a cohort of 10,624 preterm newborns (born before 37 weeks of gestational age) and showed that the vast majority of children reached a weight’s catch-up growth at 24 months of life, especially those who were SGA at birth (90.3%). Moreover, in Han et al.‘s study, catch-up growth was associated with an increased risk of being overweight in later life.^21^ On the other hand, Santos et al. highlighted a failure to thrive in the first 2 years of life for late preterm infants rather than term ones.^22^

There is no consensus regarding the proper weight gain pattern for preterm infants.^23^ Furthermore, an emerging body of evidence suggests that body composition analysis of preterm infants may help optimize infant nutrition.^24,25^ In a recent study, McLeod et al. evaluated the possibility of monitoring early changes in the body composition of preterm infants in response to specific macronutrient intake. In particular, they highlighted that higher energy and fat intake was positively associated with increased fat mass and higher protein and carbohydrate intake was positively associated with increased fat-free mass.^24,26^ Moreover, changes in the percentage of fat mass and fat-free mass in children who were preterm born are associated with different growth patterns and could influence neurological and motor long-term outcomes and be related to cardiovascular and metabolic diseases in adulthood.^24,27^ In their studies, Roggero et al. and Yau and Chang reported that preterm newborns at term corrected age showed a higher percentage of body fat content, measured by an air displacement plethysmography system than full-term infants.^27,28^ These findings were even more evident for SGA preterm neonates than in SGA term ones, as was shown by Giannì et al., whereas the mean body weight between the two study groups was comparable.^29^

Moreover, our results showed that having IUGR is associated with a greater chance of achieving catch-up growth per length at 36 months of corrected age. Several studies highlighted that children with IUGR often show a pronounced catch-up growth in the first years of life, getting closer to the genetic growth trajectory.^20,30^ On the other hand, Kesavan and Devaskar^31^ underlined that although having IUGR is associated with rapid catch-up growth in early life, the more severe IUGR is, the less these children will reach a standard height in adulthood.

In our study, among the non-modifiable factors, being born singleton represents a risk factor for slower weight and height growth velocity in early life. These data have been confirmed by several studies in the literature.^20,32^ Therefore, we could speculate that the initial IUGR and SGA births found more frequently in twins than in singletons are due to factors related to pregnancy, and a resulting catch-up growth could be expected in extrauterine life. However, the relationship between twin pregnancy and IUGR or SGA infants still needs further studies to be adequately investigated.

Despite the lack of data in the literature, the postnatal growth of late preterm infants is undoubtedly influenced by several variables, including type and mode of feeding.^14^ Although Verd et al. in their study, have evaluated the growth pattern of two groups of extremely low birth weight infants divided into exclusive human milk-fed and formula-fed one, no significant differences were found in terms of weight gain in the two groups.^33^ On the other hand, several studies have evaluated the effect of an exclusively human milk-based diet on preterm infants’ growth, and the vast majority of them found a lower percentage of fat mass deposition when compared to mainly formula-fed infants.^34–36^ Moreover, in a recent study, Mòl et al. compared a cohort of mainly formula-fed preterm infants to a group of term newborns. A more significant deposition of fat mass was highlighted in the group of preterm formula-fed babies rather than term ones^37^ and this effect could be dose-dependent, as reported by Giannì et al.^34^ Furthermore, exclusively breastfed preterm infants showed a slower weight gain than term ones.^38^ The mechanism that links breastfeeding to a slower growth pattern than formula feeding and that ultimately leads to better metabolic and neurodevelopmental outcomes was defined by Rozè et al. as an “apparent paradox of breastfeeding”.^39^ This “paradox” could be partially explained by higher fat-free mass deposition as a possible breastfeeding protective factor against the risk of obesity in adulthood.^38,40^ Similarly, in our study, we have shown a protective effect of breastfeeding on preterm infants’ growth. Indeed, infants who were any human milk-fed were protected from not achieving both weight and length catch-up growth at 36 months, showing probably a slower but better growth pattern than formula-fed ones. Regardless of these results, our study showed a very low exclusively breastfeeding rate at discharge, equal to 18%. This finding, despite being widely improvable, appeared to be quite similar to what reported by Davanzo et al. from 13 Italian NICUs.^41^ The authors classified their population based on birth weight and showed an exclusively breastfeeding rate equal to 25% and 22%, respectively, for newborns with birth weight between 1500 and 2000 g and between 2000 and 2499 g.^41^ Moreover, our population includes late preterm and SGA infants, for whom breastfeeding rates are generally lower than full-term newborns.^41,42^ In addition, 55% of our cohort were twins and 88% were born by caesarean section, both factors associated with greater difficulty in starting and continuing breastfeeding.^43,44^

Being a retrospective study, lots of data relating to the growth of the enrolled children were not available. In our opinion, the main limitations of this study are the lack of infants’ body composition assessment and a more in-depth analysis of the type of feeding and breastfeeding duration to evaluate a possible dose-dependent role of human milk on infants’ growth. Moreover, since the study analyzed a period ranging from 2009 to 2015, the evaluation of anthropometric parameters was performed using the Fenton and WHO charts and the same charts were used for both singleton and twins. Since 2014, new postnatal growth standards for preterm infants by the International Fetal and Newborn Growth Consortium for the 21st Century (INTERGROWTH 21st) have been published.^7^ It could be possible, that using different charts we could have highlighted a different growth trajectory, at least up to 6 months of corrected age.^7,45^

On the contrary, to the best of our knowledge, this is one of the few studies on assessing late preterm infants’ growth from the hospital discharge and through a long follow-up period.

Our results highlighted that late preterm and SGA infants present a variable growth trajectory during early infancy and that maintaining catch-up growth during the first year of life could be affected by different variables such as being born singleton, having IUGR, and being breastfed. Therefore, identifying these variables, especially the modifiable ones and enhancing the protective factors, is a priority for pediatricians to optimize growth and development and prevent the onset of non-communicable diseases in the short and long term. Moreover, our study confirmed the importance of breastfeeding for its nutritional value and as a protective factor against the risk of failure to thrive for preterm and SGA infants. Consequently, breastfeeding promotion should be strongly supported in neonatal intensive care among preterm infants.

Further studies with larger samples are needed to investigate the late preterm profoundly, SGA neonates’ growth trajectory and to identify possible protective factors both on weight and length gain and long-term health outcomes.

## Data Availability

All data generated and analyzed during this study are included in this published article.

## References

[1] Williams JE, Pugh Y (2018). The late preterm. Crit. Care Nurs. Clin. North Am..

[2] Delnord M, Zeitlin J (2019). Epidemiology of late preterm and early term births – an international perspective. Semin. Fetal Neonatal Med..

[3] Sharma D, Padmavathi IV, Tabatabaii SA, Farahbakhsh N (2021). Late preterm: a new high risk group in neonatology. J. Matern. Fetal Neonatal Med..

[4] Lapillonne A (2019). Feeding the late and moderately preterm infant: a position paper of the European Society for Paediatric Gastroenterology, Hepatology and Nutrition Committee on Nutrition. J. Pediatr. Gastroenterol. Nutr..

[5] Fleig L (2021). Growth outcomes of small for gestational age preterm infants before and after implementation of an exclusive human milk-based diet. J. Perinatol..

[6] Fenton TR (2003). A new growth chart for preterm babies: Babson and Benda’s chart updated with recent data and a new format. BMC Pediatr..

[7] Villar J (2014). International standards for newborn weight, length, and head circumference by gestational age and sex: the Newborn Cross-Sectional Study of the INTERGROWTH-21st Project. Lancet.

[8] González-García L (2021). Extrauterine growth restriction in very low birth weight infants: concordance between Fenton 2013 and INTERGROWTH-21st Growth Charts. Front. Pediatr..

[9] Melamed N (2021). FIGO (International Federation of Gynecology and Obstetrics) initiative on fetal growth: best practice advice for screening, diagnosis, and management of fetal growth restriction. Int J. Gynecol. Obstet..

[10] Reddy M (2021). The impact of the definition of preeclampsia on disease diagnosis and outcomes: a retrospective cohort study. Am. J. Obstet. Gynecol..

[11] WHO Multicentre Growth Reference Study (MGRS). The WHO Child Growth Standards. S3–S84 (2004).

[12] Shaker CS (2013). Cue-based feeding in the NICU: using the infant’s communication as a guide. Neonatal Netw..

[13] Shaker C (2017). Infant-guided, co-regulated feeding in the neonatal intensive care unit. Part I: theoretical underpinnings for neuroprotection and safety. Semin Speech Lang..

[14] Natarajan G, Shankaran S (2016). Short- and long-term outcomes of moderate and late preterm infants. Am. J. Perinatol..

[15] Baldassarre ME (2022). Complementary feeding in preterm infants: a position paper by Italian Neonatal, Paediatric and Paediatric Gastroenterology Joint Societies. Ital. J. Pediatr..

[16] Fanaro S, Borsari G, Vigi V (2007). Complementary feeding practices in preterm infants: an observational study in a cohort of Italian infants. J. Pediatr. Gastroenterol. Nutr..

[17] Morgan JB, Williams P, Foote KD, Marriott LD (2006). Do mothers understand healthy eating principles for low-birth-weight infants?. Public Health Nutr..

[18] Van IJzendoorn MH, Bakermans-Kranenburg MJ, Juffer F (2007). Plasticity of growth in height, weight, and head circumference: meta-analytic evidence of massive catch-up after international adoption. J. Dev. Behav. Pediatrics.

[19] Ghods, E., Kreissl, A., Brandstetter, S., Fuiko, R. & Widhalm, K. Head circumference catch-up growth among preterm very low birth weight infants: effect on neurodevelopmental outcome. *J. Perinat. Med.* **39**, 579–586 (2011). 10.1515/jpm.2011.049 21740330

[20] Zhang L (2019). Postnatal length and weight growth velocities according to Fenton reference and their associated perinatal factors in healthy late preterm infants during birth to term-corrected age: an observational study. Ital. J. Pediatr..

[21] Han J (2021). Postnatal growth of preterm infants during the first two years of life: catch-up growth accompanied by risk of overweight. Ital. J. Pediatr..

[22] Santos IS (2009). Late preterm birth is a risk factor for growth faltering in early childhood: a cohort study. BMC Pediatr..

[23] Villar J (2018). Monitoring the postnatal growth of preterm infants: a paradigm change. Pediatrics.

[24] Hamatschek C (2020). Fat and fat-free mass of preterm and term infants from birth to six months: a review of current evidence. Nutrients.

[25] Bortolotto CC (2021). Prematurity and body composition at 6, 18, and 30 years of age: Pelotas (Brazil) 2004, 1993, and 1982 birth cohorts. BMC Public Health.

[26] McLeod G (2015). Feasibility study: assessing the influence of macronutrient intakes on preterm body composition, using air displacement plethysmography: preterm nutrition and body composition. J. Paediatr. Child Health.

[27] Roggero P (2012). Growth and fat-free mass gain in preterm infants after discharge: a randomized controlled trial. Pediatrics.

[28] Tsou Yau K-I, Chang M-H (1993). Growth and body composition of preterm, small-for-gestational-age infants at a postmenstrual age of 37–40 weeks. Early Hum. Dev..

[29] Giannì ML (2012). Body composition in newborn infants: 5-year experience in an Italian neonatal intensive care unit. Early Hum. Dev..

[30] Ong KKL (2000). Association between postnatal catch-up growth and obesity in childhood: prospective cohort study. BMJ.

[31] Kesavan K, Devaskar SU (2019). Intrauterine growth restriction. Pediatr. Clin. North Am..

[32] Morag I (2018). Postnatal growth disadvantage of the small for gestational age preterm twins. Nutrients.

[33] Verd S (2015). Hospital outcomes of extremely low birth weight infants after introduction of donor milk to supplement mother’s milk. Breastfeed. Med..

[34] Giannì M (2016). Does human milk modulate body composition in late preterm infants at term-corrected age?. Nutrients.

[35] Piemontese P (2018). The effect of human milk on modulating the quality of growth in preterm infants. Front. Pediatr..

[36] Olhager, E. & Törnqvist, C. Body composition in late preterm infants in the first 10 days of life and at full term. *Acta. Paediatr.* **103**, 737–743. 10.1111/apa.12632 (2014). 10.1111/apa.12632 24628453

[37] Mól N, Zasada M, Kwinta P (2019). Does type of feeding affect body composition in very low birth weight infants? – A prospective cohort study. Pediatr. Neonatol..

[38] Brownell EA (2018). Dose-response relationship between donor human milk, motherʼs own milk, preterm formula, and neonatal growth outcomes. J. Pediatr. Gastroenterol. Nutr..

[39] Rozé J-C (2012). The apparent breastfeeding paradox in very preterm infants: relationship between breast feeding, early weight gain and neurodevelopment based on results from two cohorts, EPIPAGE and LIFT. BMJ Open.

[40] Scientific Center of Children’s Health, Moscow, Russian Federation. (2014). Peculiarities of physical growth and body composition of preterm infants, received different types of feeding, at the discharge from hospital. ARAMS.

[41] Davanzo R (2013). Breastfeeding at NICU discharge: a multicenter Italian study. J. Hum. Lact..

[42] Jonsdottir RB, Jonsdottir H, Orlygsdottir B, Flacking R (2021). A shorter breastfeeding duration in late preterm infants than term infants during the first year. Acta Paediatr..

[43] Zhang F, Cheng J, Yan S, Wu H, Bai T (2019). Early feeding behaviors and breastfeeding outcomes after cesarean section. Breastfeed. Med..

[44] Quitadamo, P., Comegna, L. & Cristalli, P. Anti-infective, anti-inflammatory, and immunomodulatory properties of breast milk factors for the protection of infants in the pandemic from COVID-19. *Front. Public Health* **8**, 589736 (2021). 10.3389/fpubh.2020.589736 PMC7960784 33738273

[45] Giorgione V, Briffa C, Di Fabrizio C, Bhate R, Khalil A (2021). Perinatal outcomes of small for gestational age in twin pregnancies: twin vs. singleton charts. J. Clin. Med..

